# Analysis of Imported Cases of COVID-19 in Taiwan: A Nationwide Study

**DOI:** 10.3390/ijerph17093311

**Published:** 2020-05-09

**Authors:** Jui-Yao Liu, Tzeng-Ji Chen, Shinn-Jang Hwang

**Affiliations:** 1 Department of Family Medicine, Taipei Veterans General Hospital, No.201, Sec. 2, Shi-Pai Road, Taipei 11217, Taiwan; ryliu@vghtpe.gov.tw (J.-Y.L.); sjhwang@vghtpe.gov.tw (S.-J.H.); 2 School of Medicine, National Yang-Ming University, No.155, Sec. 2, Linong Street, Taipei 11217, Taiwan; 3 Big Data Center, Department of Medical Research, Taipei Veterans General Hospital, No. 201, Sec. 2, Shi-Pai Road, Taipei 11217, Taiwan

**Keywords:** 2019 novel coronavirus diseases (COVID-19), pandemic outbreak, imported cases, border control, quarantine, isolation, contact tracing, symptom, reproduction number

## Abstract

In the early stages of the 2019 novel coronavirus disease (COVID-19) pandemic, containment of disease importation from epidemic areas was essential for outbreak control. This study is based on publicly accessible data on confirmed COVID-19 cases in Taiwan extracted from the Taiwan Centers for Disease Control website. We analysed the characteristics, infection source, symptom presentation, and route of identification of the 321 imported cases that were identified from 21 January to 6 April 2020. They were mostly returned Taiwanese citizens who had travelled to one or more of 37 countries for tourism, business, work, or study. Half of these cases developed symptoms before arrival, most of the remainder developed symptoms 1–13 days (mean 4.0 days) after arrival, and 3.4% never developed symptoms. Three-quarters of the cases had respiratory symptoms, 44.9% had fever, 13.1% lost smell or taste, and 7.2% had diarrhoea. Body temperature and symptom screening at airports identified 32.7% of the cases. Of the remainder, 27.7% were identified during home quarantining, 16.2% were identified via contact tracing, and 23.4% were reported by hospitals. Under the strict enforcement of these measures, the incidence of locally acquired COVID-19 cases in Taiwan remains sporadic. In conclusion, proactive border control measures are effective for preventing community transmission of this disease.

## 1. Introduction

The 2019 novel coronavirus disease (COVID-19) caused by severe acute respiratory syndrome (SARS) coronavirus 2 (SARS-CoV-2) that emerged in Wuhan, China in December 2019 was declared a global pandemic on 11 March 2020 by the World Health Organization [1]. As of 29 April 2020, the disease has spread to 185 countries, with 3,114,659 confirmed cases and 216,989 deaths [2]. The case fatality rate is about 6.9% globally, but mortality in elderly patients with comorbidities is higher [3,4,5]. Under the current circumstances, in which there is no vaccine available for prevention and no effective antiviral drug for treatment, almost all people in the world are susceptible to this novel person-to-person transmitted disease [5]. The complexity and high volume of international air travel has allowed the disease to spread rapidly [6,7,8]. In the early stages of this pandemic, containment of disease importation from epidemic areas was essential for preventing indigenous outbreaks [9]. Without adequate control measures, persistent and imperceptible virus importation via air travellers may cause large-scale community transmission. This could lead to an outbreak that may exceed the capacity of the healthcare system with disastrous results [10]. 

The Taiwanese authorities have made efforts to contain the importation of the disease by issuing travel advisories and implementing flight bans, entry restrictions, airport screening, home quarantining of travellers from high-risk areas, isolation of confirmed cases, and thorough contact tracing [11,12,13]. The Taiwan Centers for Disease Control (CDC) established fever screening sites with infrared thermal imaging cameras in international airports in 2003 following the SARS outbreak of that year. During the COVID-19 outbreak, they have been performing airport screening at these fever screening sites. Travellers entering Taiwan with fever or respiratory symptoms must have oropharyngeal specimens collected for COVID-19 testing and are subject to home quarantining for 14 days, as are travellers from areas where the disease is epidemic. People in home quarantine must stay at home (or at another designated location) and not go out, and they must maintain a distance of at least one metre from their family, record body temperature and health status every day, and cooperate with tracking measures implemented by their local borough chief. Individuals who develop symptoms such as fever, coughing, or runny nose while in home quarantine are sent to designated medical facilities for COVID-19 testing. When COVID-19 cases are confirmed, they are immediately hospitalized in negative pressure isolation rooms, and Taiwan CDC personnel conduct investigations within 24 h to identify their contacts. Any contacts who have COVID-19-related symptoms are hospitalized for testing. Contacts who do not show symptoms or who test negative for COVID-19 are placed in home isolation for 14 days for further follow-up and testing. People in home isolation must stay at home (or other designated location) and not go out, similarly to those in home quarantines. The local health authority checks their health status twice daily, and symptomatic individuals are sent to hospital for medical attention and COVID-19 testing. 

The approach of the Taiwanese authorities appears to have been successful in the outbreak control, it is nevertheless important to understand the characteristics of the imported cases of the disease to know how it works. The aim of this study is to analyse the imported cases of COVID-19 in Taiwan to assess their basic demographic characteristics, disease source, symptom presentation, and routes of identification.

## 2. Materials and Methods

### 2.1. Data Collection

Open-access data and press releases concerning COVID-19 in Taiwan that were available on the website of the Taiwan CDC were collected [14]. The press releases, which included attached files, provided detailed information about the confirmed COVID-19 cases in Taiwan, including their category (imported or locally acquired), citizenship, sex, age group, travel history, date of arrival, reason for travel, date of disease onset, date of specimen collection for COVID-19 testing, date of disease confirmation, and route of identification. The Taiwan CDC listed COVID-19 as a notifiable disease on 15 January 2020. This meant that hospitals and clinics had to notify the health authorities if patients were diagnosed with COVID-19 and provide their travel details, contact histories and clinical presentation, as well as send specimens (throat swab and sputum) to the Taiwan CDC for confirmation via reverse transcription polymerase chain reaction (RT-PCR). The date of disease onset was defined as the date of symptom onset. The countries or areas the imported cases had visited within 14 days prior to disease onset were defined as the source of their infection. All imported COVID-19 cases confirmed in Taiwan from 21 January to 6 April 2020 were included in this study.

### 2.2. Statistics

Descriptive statistics and plots of age group, sex, infection source, days from arrival to symptom onset, days from arrival to disease confirmation, and routes of identification were performed using PASW Statistics 18 (SPSS, Chicago, IL, USA). One-way analysis of variance (ANOVA) was used to compare effective reproduction number (*R*) and mean number of days from arrival to disease confirmation between routes of identification, using Bonferroni post hoc tests and 95% confidence intervals (CI). The case fatality rate of the imported cases was compared with that of the locally acquired cases using a chi-squared test. A two-tailed *p*-value of < 0.05 was considered statistically significant. 

## 3. Results

### 3.1. Basic Characteristics, Infection Source, and Reason for Travel 

**Figure 1:**
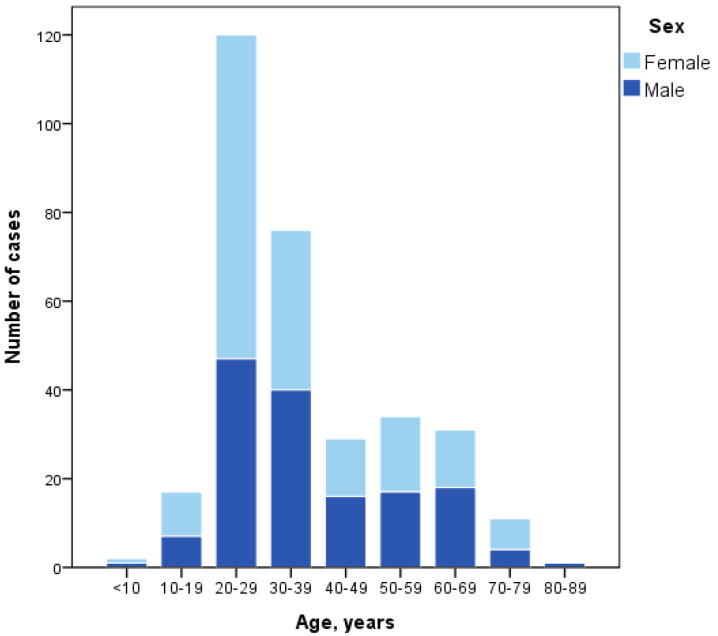
Age and sex distribution of the imported cases of COVID-19 in Taiwan from 21 January to 6 April 2020.

There was a cumulative total of 373 confirmed cases of COVID-19 in Taiwan from 21 January to 6 April 2020, 321 (86.1%) of which were imported. Of the imported cases, 96.6% were Taiwanese and 53.0% were female. Their age range was 4–88 years; young people occupied a large proportion. Of them, 37.4% were in age group of 20–29 years, 23.7% were in age group of 30–39 years (Figure 1). The main reasons for travel were tourism (104, 32.4%), business or work (88, 27.4%), study (85, 26.5%), family visit (17, 5.3%), or residency (11, 3.4%) (Table 1).

**Figure 2:**
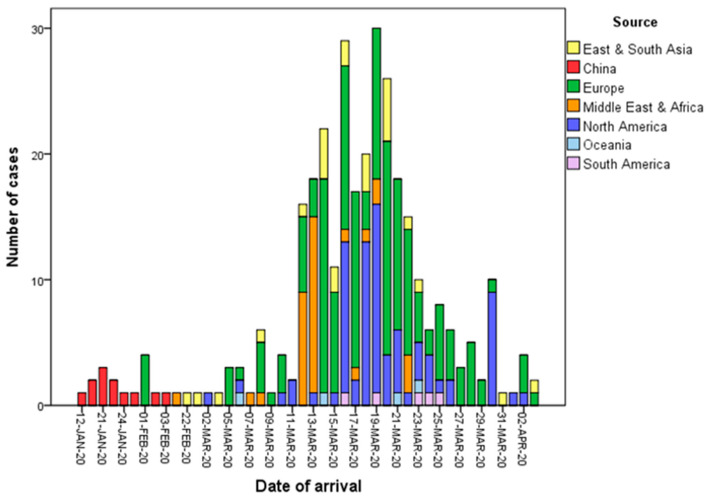
Date of arrival and infection source of imported cases of COVID-19 in Taiwan from 21 January to 6 April 2020.

The first 11 confirmed imported cases were all from Wuhan, China. The other imported cases were from East and South Asia (8.4%), the Middle East and Africa (11.0%), Europe (51.0%), North America (26.8%), South America (1.9%), and Oceania (1.3%) (Figure 2). Of the cases aged 10–19 and 20–29 years, 76.5% and 53.3%, respectively, were studying abroad. They returned home because their educational institutions were closed in response to the outbreak. There were some instances of several cases who were studying at the same college in the United Kingdom or Spain, where clusters of infections occurred on campus. There were also several clusters of cases from tour groups that were travelling in Egypt, Turkey, or Europe. An aeroplane that flew from New York to Taipei on 30 March 2020 carried 12 passengers who were subsequently confirmed to be infected with COVID-19. 

### 3.2. Symptom Presentation

Only 44.9% of the cases had fever. A large majority (73%) of the cases had respiratory symptoms, comprising cough (45.5%), sore throat (31.2%), rhinorrhoea or nasal stuffiness (29.9%), chest tightness or pain (5.6%), and dyspnea (3.4%). Some cases had flu-like symptoms, such as malaise (16.2%), myalgia or arthralgia (12.5%), or headache (10.6%). A proportion of the cases (13%) had the neurological symptoms of loss of smell or taste. Few of the cases (8%) had gastrointestinal symptoms, specifically diarrhoea (7.2%), nausea or vomiting (0.9%), and abdominal pain (0.9%), and even fewer (2%) had ophthalmic symptoms such as itching, congestion, or pain in the eyes. Eleven cases (3.4%) did not have any symptoms (Table 2).

**Figure 3:**
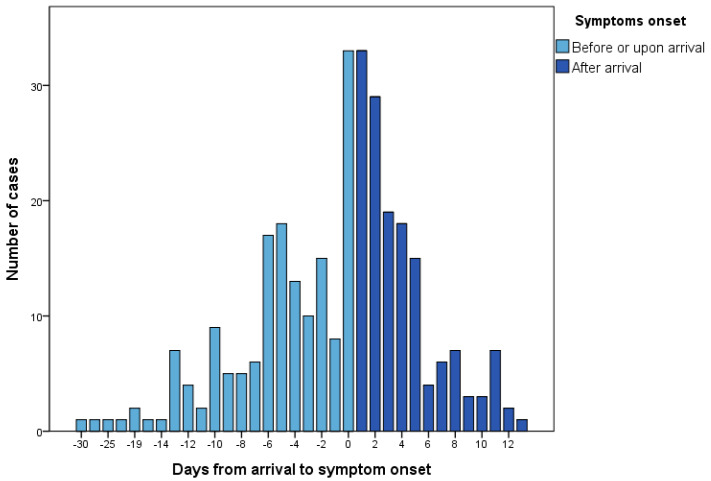
Time from arrival to symptom onset in imported COVID-19 cases in Taiwan from 21 January to 6 April 2020.

About half (50.5%) of the imported cases had developed symptoms before arrival (mean 5.4 days, range 0–30 days before arrival) (Figure 3). Of the cases who did not display symptoms on arrival, most (93.1%) developed symptoms 1–13 days after arrival (mean 4.0 days, median three days) (Figure 3). Eleven of the imported cases did not develop any symptoms; these were identified via contact testing.

### 3.3. Identification Routes

**Figure 4:**
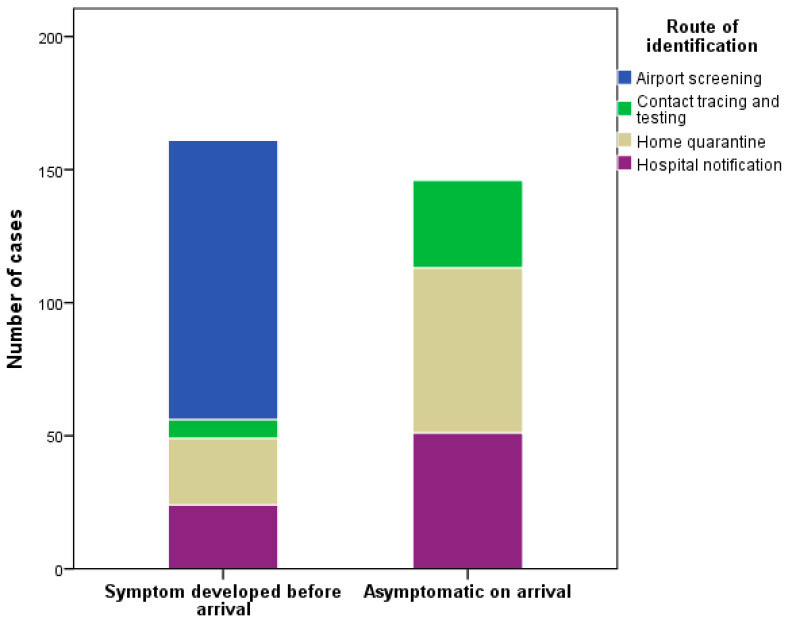
The route of identification of imported COVID-19 cases in Taiwan from 21 January to 6 April 2020, stratified by time of symptom onset relative to arrival.

Of the imported cases, 32.7% were identified in airport screening, 27.7% during home quarantine, 16.2% through contact tracing, and 23.4% sought medical attention themselves and were reported by the hospitals (Table 3). Almost two-thirds (64.8%) of the cases who had developed symptoms before arrival were identified in airport screening. Of the cases who were asymptomatic on arrival, 39.6% were identified during home quarantine, 28.3% were identified through contact tracing, and 32.1% were reported by hospitals (Figure 4). 

### 3.4. Extension of Quarantine Measure after COVID-19 Declared Global Pandemic

**Figure 5:**
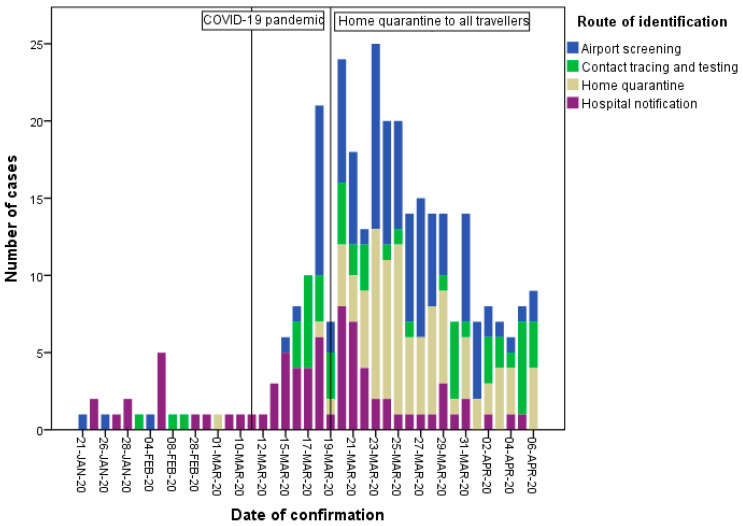
Number of imported COVID-19 cases in Taiwan from 21 January to 6 April 2020, stratified by date of confirmation and route of identification.

The number of imported cases increased dramatically after 11 March 2020, when COVID-19 was declared a global pandemic (Figure 5). The mandatory 14-day home quarantine was extended to all travellers from all countries on 19 March 2020. This measure kept all travellers from high-risk areas confined to their homes under close monitoring, and prevented them from moving around in their communities. After implementation of this measure, most of the imported cases were contained in home quarantine or home isolation before they were identified (Figure 5).

### 3.5. Time between Arrival and Disease Confirmation 

**Figure 6:**
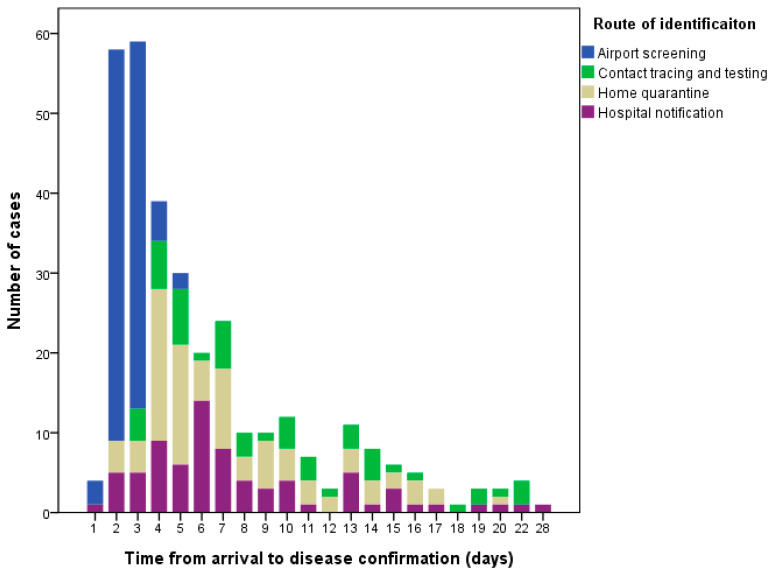
Time from arrival to disease confirmation for imported COVID-19 cases in Taiwan from 21 January to 6 April 2020.

The time from arrival to disease confirmation of the imported cases was 1–28 days (mean 6.3 days) (Figure 6). Few of the cases identified during home quarantining had longer than 14 days of time from arrival to disease confirmation because they delayed reporting their symptoms or had borderline COVID-19 test results, which necessitated repeat sampling and testing for confirmation. The cases identified through airport screening had the shortest time from arrival to disease confirmation (mean 2.6 days, 95% CI: 2.4–2.7 days, *p* < 0.01) as compared to cases identified via the other routes (Table 3). The time from symptom onset to disease confirmation was 1–32 days (mean 7.0 days). 

### 3.6. Locally Acquired Cases Infected by Imported Cases

**Figure 7:**
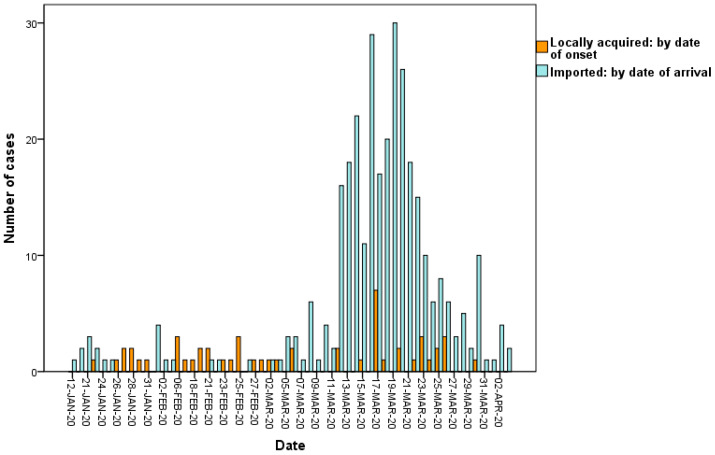
Number of confirmed cases of COVID-19 in Taiwan up to 6 April 2020.

The incidence of locally acquired COVID-19 cases remains sporadic although there are hundreds of imported COVID-19 cases in Taiwan (Figure 7). Nineteen of the 52 (36.5%) locally acquired cases were infected by 16 of the 321 imported cases, yielding an *R* of 0.06 for all imported cases and 1.2 for the 16 imported cases. These were family, friends, colleagues, and classmates of the imported cases. No locally acquired cases were infected by the cases who were identified via airport screening. The *R* was significantly related to the route of identification (*p* < 0.05; Table 3). That of the cases identified via contact tracing was significantly higher than that of the cases identified through airport screening (0.15 versus 0, *p* < 0.05; Table 3). Three of the 321 imported cases and three of the 52 locally acquired cases had died. The case fatality rate of the imported cases was significantly lower than that of the locally acquired cases (0.9% versus 5.8%, *p* = 0.01).

## 4. Discussion

There were two waves of COVID-19 importation in Taiwan. The first wave was from China, and was well contained by early preventive measures. The Taiwan CDC performed onboard inspection for all direct flights arriving from Wuhan since 31 December 2019. Over the next few weeks, they gradually expanded the range of restrictions on travel to China and implemented the 14-day home quarantine for travellers from China after Wuhan went into lockdown. The second wave of COVID-19 importation came from 36 other countries around the world, mainly the United States, the United Kingdom, and several other European countries. Inspection of the arrival dates of the second wave of imported cases reveals that these arrivals in Taiwan were ahead of the timing of COVID-19 outbreak in the other countries. The first cases imported from Italy arrived on 1 February, when there were only two confirmed cases in Italy [2]. These were four members of a Taiwanese family that travelled to Italy, with a transfer at the airport in Hong Kong, from 22 January to 1 February. Three of the family members developed symptoms of cough or fever during the journey. There may have been many as-yet undiscovered cases of COVID-19 in Italy at that time. The mandatory 14-day home quarantine was extended to travellers from Italy on 27 February, from other European countries on 14 March, from the Middle East and Africa on 16 March, from East and South Asia on 17 March, and finally to travellers from all countries on 19 March. The imported cases identified in home quarantine seemed to have a lower *R* than those identified via hospital notification or contact tracing (Table 3). However, some individuals in home quarantine did not adhere to the instructions to stay at home, which would have exposed otherwise-unaffected people to the risk of infection [15,16].

Airport screening of body temperature and respiratory symptoms was able to detect 32.7% of the imported COVID-19 cases and 64.8% of those who had developed symptoms before arrival. The remaining cases who had developed symptoms before arrival evaded detection because they had taken antipyretic drugs, did not honestly declare their symptoms, or their symptoms were mild or not involving the respiratory tract. In fact, travellers with any suspicious symptoms are able to notify the airport health-screening personnel and be tested for COVID-19. Nevertheless, airport screening is an effective measure to identify symptomatic imported cases soon after their arrival.

There was clustering of the imported COVID-19 cases among household members, members of the same tour group, passengers on the same aeroplane, and even students from the same overseas campus. The cases identified via contact tracing had a higher *R* and a longer interval between arrival and disease confirmation than those identified via other routes (Table 3). The cases who never developed symptoms were difficult to identify without testing the contacts of confirmed cases. Thus, thorough contact tracing and testing is important for interrupting all possible transmission chains [17]. 

Taiwan has several advantages in terms of outbreak control. First, more than 99.9% of the population is enrolled in the National Health Insurance (NHI) program. Individuals with a history of travel and suspicious symptoms have therefore been willing to seek medical attention because the costs of COVID-19 testing and treatment are covered by the government. Second, the NHI smart card stores information of border entry and exit records and the home quarantine or isolation status of the insured individual, which alerts medical personnel to high-risk patients. Third, NHI claim data can provide a list of symptomatic patients who sought medical attention after returning from high-risk countries, which can be used to implement quarantine measures and retrospective contact tracing. 

Most of the COVID-19 cases who have moderate to severe disease present with fever [18], but half of our imported COVID-19 cases did not have a fever. Body temperature screening at the airport did not detect all cases, and in particular missed those without obvious symptoms. Most of our imported cases had mild disease and would have been difficult to identify if their travel and contact history had not been available [19]. In addition to respiratory symptoms, some cases had neurological symptoms, such as loss of smell or taste [20], or gastrointestinal symptoms like diarrhoea. The clinical presentations of COVID-19 involve multi-organ systems not limited to the respiratory tract. Some imported cases had long duration of symptoms display before arrival, the longest in this study was 30 days, and still could be tested positive for SARS-CoV-2 nucleic acid by RT-PCR. Prolonged viral shedding [3], a long transmissibility period, and the fact that asymptomatic or paucisymptomatic patients can transmit this disease make the disease control challenging [21,22,23].

## 5. Conclusions

Proactive border control measures to contain the importation of COVID-19 via airport screening, quarantining of travellers from epidemic areas, and thorough contact tracing are effective for preventing community transmission of this disease. Under the strict enforcement of these measures, the incidence of locally acquired COVID-19 cases in Taiwan remains sporadic. 

## Figures and Tables

**Table 1:** Confirmed imported cases of COVID-19 in Taiwan from 21 January to 6 April 2020, sorted according to the date of arrival. (Note that there is some duplication because some cases travelled to multiple countries during their incubation period).

Source	No. of Cases (N = 321) (%)	Date of Arrival	Date of Confirmation	Reasons for Travel	
				Taiwanese (n = 310)	Non-Taiwanese (n = 11)
China (Wuhan)	11 (3.4)	12/1–3/2	21/1–6/2	business, tourism, residency	tourism
Macao	1 (0.3)	24/1	6/2	tourism	
Italy	7 (2.2)	1/2–19/3	6/2—24/3	tourism, study, business	
Egypt	12 (3.7)	21/2–19/3	29/2–30/3	tourism	
Dubai	10 (3.1)	21/2–13/3	29/2–17/3	tourism	
Japan	2 (0.6)	22/2–8/3	28/2–15/3	tourism	
United States	81 (25.2)	2/3–2/4	18/3–6/4	tourism, study, residency, business, family visit, conference, work	business, work
Philippines	11 (3.4)	3–23/3	5–31/3	tourism, family visit, study, business, work	
Greece	1 (0.3)	5/3	15/3	tourism	
Netherlands	8 (2.5)	5–20/3	10–25/3	business, tourism, study	
Germany	10 (3.1)	6–21/3	14/3–4/4	business, tourism, sporting event, work	
Mexico	2 (0.6)	6/3–21/3	20/3–26/3	tourism	
Australia	2 (0.6)	6/3–23/3	27/3–28/3	work, business	
United Kingdom	73 (22.7)	8/3–4/4	11/3–6/4	tourism, study, business, sporting event, residency, work, religion	
Belgium	4 (1.2)	8–20/3	12–25/3	tourism, study	
Switzerland	6 (1.9)	8–20/3	14–31/3	tourism, study, business	
France	21 (6.5)	8–27/3	14–31/3	tourism, business, study, work	tourism, family visit
Turkey	18 (5.6)	8–20/3	15–29/3	tourism	
Thailand	6 (1.9)	8–20/3	15/3–3/4	tourism, study	
Portugal	2 (0.6)	9/3–16/3	20/3–29/3	tourism	
Ireland	4 (1.2)	9–20/3	12–28/3	tourism, study, business	
Spain	17 (5.3)	9–27/3	15–29/3	tourism, study	
Austria	10 (3.1)	10–14/3	14/3–5/4	tourism	work
Iceland	4 (1.2)	10–28/3	17/3–6/4	tourism	
Indonesia	8 (2.5)	12/3–4/4	18/3–6/4	tourism, work	wedding, work
Czech Republic	15 (4.7)	14–20/3	16/3–5/4	tourism, work, study, business	
New Zealand	2 (0.6)	14/3–21/3	22/3–26/3	tourism	
Poland	1 (0.3)	15/3	20/3	study tour	
Denmark	2 (0.6)	15/3	22/3–2/4	family visit	
Qatar	2 (0.6)	16/3–19/3	18/3–25/3	business	
Countries in South America	5 (1.6)	16–25/3	23/3–4/4	tourism	
Canada	2 (0.6)	16/3–26/3	20/3–28/3	tourism, study	
Malaysia	1 (0.3)	16/3	26/3	business	
Luxembourg	1 (0.3)	17/3	20/3	tourism	
Singapore	1 (0.3)	18/3	20/3	business	
South Africa	1 (0.3)	18/3	21/3	business	
Monaco	3 (0.9)	22/3	26/3–5/4	tourism	
Tunisia	1 (0.3)	27/3	29/3	study tour	

**Table 2:** Symptom presentation of the 321 imported COVID-19 cases in Taiwan from 21 January to 6 April 2020.

Category and Presentation	n	%
Generalised symptoms		
Fever	144	44.9
Chills	9	2.8
Malaise	52	16.2
Myalgia or arthralgia	40	12.5
Respiratory symptoms	233	72.6
Cough	146	45.5
Sore throat	100	31.2
Rhinorrhoea, sneezing, nasal stuffiness	96	29.9
Chest tightness or pain	18	5.6
Dyspnea	11	3.4
Neurological symptoms	76	23.7
Loss of smell or taste	42	13.1
Headache	34	10.6
Dizziness	6	1.9
Gastrointestinal symptoms	26	8.1
Diarrhoea	23	7.2
Abdominal pain	3	0.9
Nausea or vomiting	3	0.9
Ophthalmic symptoms		
Itching, congestion, or pain in the eyes	6	1.9
No symptoms	11	3.4

**Table 3:** Reproduction number (*R*) and time from arrival to disease confirmation of the 321 imported COVID-19 cases in Taiwan from 21 January to 6 April 2020, stratified by route of identification.

Route of Identification	n (%)	No. of Locally Acquired Cases as Their Infectees	*R* * (95% CI)	Days from Arrival to Disease Confirmation Mean (95% CI)
Airport screening	105 (32.7)	0	0 (0–0) **	2.6 (2.4–2.7) ***
Home quarantine	89 (27.7)	4	0.04 (0.00–0.09)	7.4 (6.5–8.3)
Contact tracing	52 (16.2)	8	0.15 (0.00–0.30)	9.7 (8.2–11.3)
Hospital notification	75 (23.4)	7	0.09 (0.02–0.17)	7.8 (6.7–9.0)

* *p* < 0.05 between identification routes by one-way ANOVA, ** *p* < 0.05 by Bonferroni post hoc test, compared to cases identified through contact tracing, *** *p* < 0.01 by Bonferroni post hoc test, compared to cases identified via the other three routes.
